# Developmental origins of diversity in cerebellar output nuclei

**DOI:** 10.1186/1749-8104-9-1

**Published:** 2014-01-09

**Authors:** Mary J Green, Richard JT Wingate

**Affiliations:** 1 MRC Centre for Developmental Neurobiology, King’s College London, 4th floor New Hunt’s House, London SE1 1UL UK; 2 MRC National Institute for Medical Research, The Ridgeway, Mill Hill, London NW7 1AA UK

**Keywords:** Chick, Mouse rhombic lip, Atoh1, Lhx9

## Abstract

**Background:**

The functional integration of the cerebellum into a number of different neural systems is governed by the connection of its output axons. In amniotes, the majority of this output is mediated by an evolutionarily diverse array of cerebellar nuclei that, in mice, are derived from the embryonic rhombic lip. To understand the origins of cerebellar nucleus diversity, we have explored how nucleus development is patterned in birds, which notably lack a dentate-like nucleus output to the dorsal thalamus.

**Results:**

Using targeted *in ovo* electoroporation of green fluorescent protein (GFP) and red fluorescent protein (RFP) in a variety of combinations and with different conditional enhancers, we show that cerebellar nuclei in chicks are produced, as in the mouse, at the rhombic lip. Furthermore, the comparison of fate-mapped neurons with molecular markers reveals a strict temporal sequence of cell fate allocation in establishing the avian lateral and medial cerebellar nuclei. In contrast to the mouse cerebellum, *Lhx9* expression is confined to extracerebellar thalamic afferent nuclei corresponding to the absence, in chicks, of a dentate nucleus. Spatiotemporally targeted over-expression of *Lhx9* in chick cerebellar nuclei (recapitulating in part the mammalian expression pattern) results in a loss of distinct nuclear boundaries and a change in axon initial trajectories consistent with a role for *Lhx9* specifying targeting.

**Conclusions:**

Our results confirm the relationship between cell fate and a fine grain temporal patterning at the rhombic lip. This suggests that the lack of a cerebellar output to the dorsal thalamus of birds corresponds with a restricted expression of the LIM-homeodomain gene *Lhx9* to earlier born rhombic lip cohorts when compared to mice. The evolution of cerebellar nucleus diversity in amniotes may hence reflect a heterochronic adaptation of gene expression with respect to the sequential production of rhombic lip derivatives resulting in altered axonal targeting.

## Background

The cerebellum has a highly stereotyped neuronal architecture that is integrated into the function of a variety of systems via long-range axon connections. While cerebellar size and foliation varies between species, it is this connectivity, mediated for the most part by the cerebellar nuclei, that determines the range of motor, sensory and cognitive functions modulated by the cerebellum. Both number and targets of cerebellar nuclei, which are sometimes referred to as “deep” nuclei, vary among species. This ranges from none in teleosts, which instead possess eurydendroid cells of equivalent function, to one in amphibians, two in reptiles and birds and between three and five in mammals. Most significantly, the dentate nucleus, which directs cerebellar output to the dorsal thalamus and, hence, is the likely substrate for a range of higher cognitive cerebellar functions [1,2], is a mammalian innovation [3].

Relatively little is known about the development of cerebellar nuclei. While the role of a nuclear transitory zone (NTZ) has been recognised as an assembly point for immature neurons of the cerebellar nuclei since the 1980s [4], the dual origin of neurons from the rhombic lip and cerebellar ventricular zone has only more recently been revealed by genetic fate-maps. Glutamatergic projection neurons of the cerebellar nuclei are one cohort in a sequence of tangentially migrating neurons derived from an *Atoh1*-positive pool of rhombic lip precursors, which also give rise to both extra-cerebellar neurons and cerebellar granule neurons [5-7]. GABAergic interneurons of the cerebellar nuclei are born from a distinct progenitor pool in the cerebellar ventricular zone and integrate with the glutamatergic neurons in the NTZ following radial migration [8]. Fate-mapping studies suggest that early born derivatives of the rhombic lip express the transcription factor *Lhx9*, while later-born populations express *Barhl1*[9,10] and *Pax6*[11,12]. Significantly, thalamic connections appear to be restricted to neurons, including the dentate nucleus, that express *Lhx9*[9], one of a class of LIM-homeodomain proteins for which gain-of-function studies indicate a role in specifying axon trajectory [13-16].

To investigate the correlation between birth date, *Lhx9* expression and connectivity in specifying cerebellar nuclei, we investigated their patterning in chicks and mice. Birds appear to possess homologues of the mammalian fastigial and interposed nuclei, but lack a dentate nucleus and, hence, a cerebellar output to the dorsal diencephalon [17]. Using a combination of spatiotemporal targeted cell labelling, gene expression analysis and overexpression studies we show that this difference might be attributed to a shift in the temporal allocation of *Lhx9* within the rhombic lip lineage. Our observations suggest that heterochronic adaptation of the rhombic lip lineage provides an adaptive substrate for cerebellum functional evolution.

## Results

### Targeted electroporation accurately labels derivatives of the chick rhombic lip

**Figure 1:**
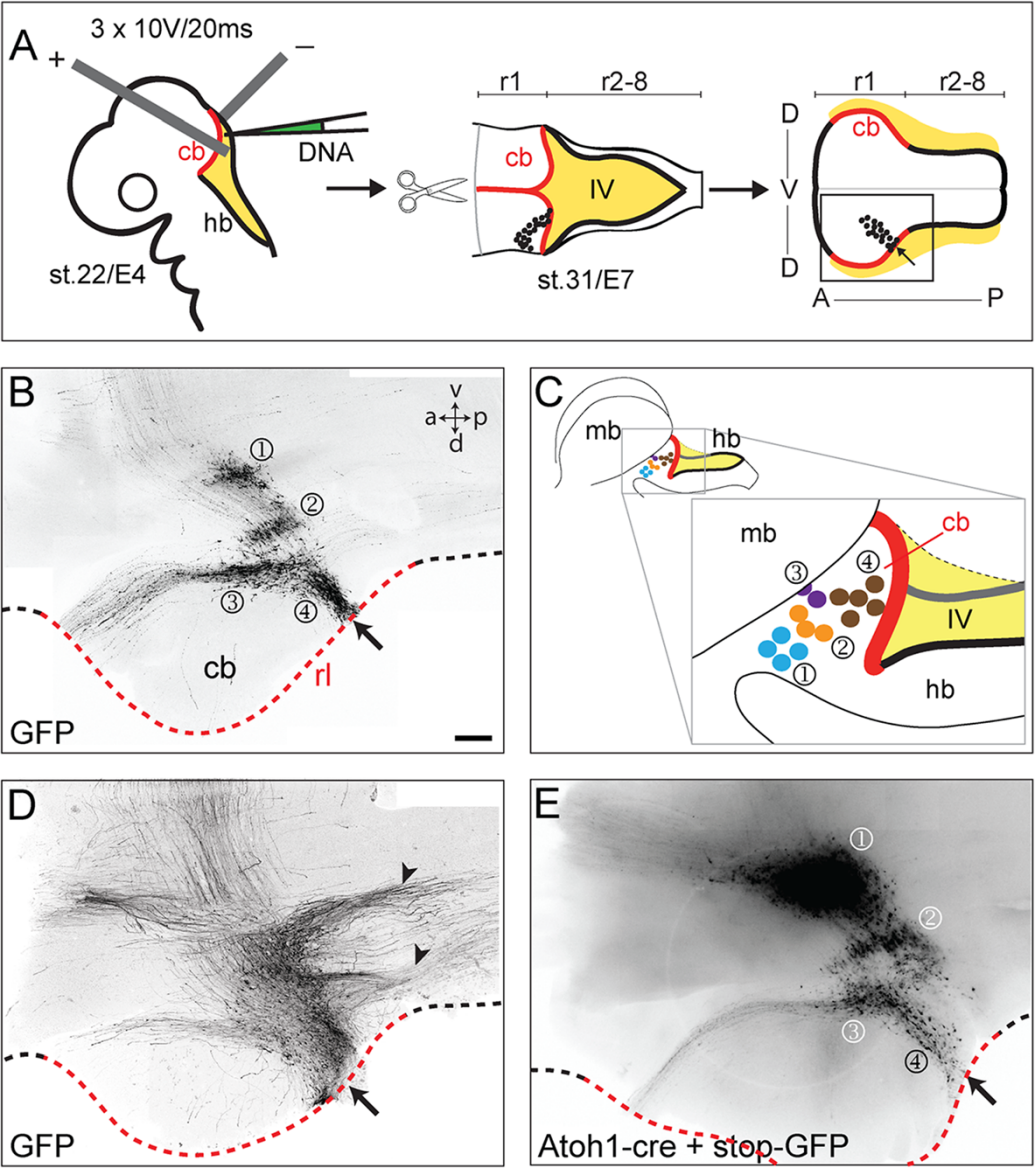
**Four populations are labelled in rhombic lip electroporations. A**. Schematic diagram of electroporation and flat-mount preparation where rhombic lip of cerebellum (cb) in red and hindbrain (hb) in black borders the fourth ventricle (IV) roof plate (yellow). **B**. Four populations of neurons (①②③④) are labelled at st.31/E7 following green fluorescent protein (GFP) electroporation at st.22/E4. Site of electroporation is indicated by arrows. Rhombic lip (rl) of cb shown by red dashed line. **C**. Positions of each population are summarised in schematic diagram of the intact midbrain/hindbrain region. **D**. In a subset of experiments, GFP was detected in additional axon tracts (arrowheads). **E**. Electroporation of Atoh1-cre + stop-GFP into cerebellar rhombic lip confirms that infrequently labelled, additional axons in **D** are not rhombic lip derived. Dashed line highlights the rhombic lip with red region indicating cerebellar rhombic lip. Arrows **(B-E)** show site of electroporation. r, rhombomere; a, anterior; p, posterior; d, dorsal; v, ventral. In this and subsequent figures, AP axis runs left to right. Scale Bars: 200 μm in **B**, **D** and **E**.

We focally electroporated the cerebellar rhombic lip (rhombomere (r)1) in chicks (n = 90) with pCAβ-green fluorescent protein (GFP) at Hamburger and Hamilton stage (st.)22 (embryonic day (E) 4) to map its derivatives and harvested embryos at st.31 (E7). Hindbrains were dissected and flat-mounted by opening the neural tube along the dorsal midline, pial surface uppermost (Figure 1A). Tangentially migrated unipolar neurons labelled with GFP over the course of three days were found in four distinct populations (Figure 1B): one in ventral r1 (Figure 1B ①); two at the lateral edge of the cerebellar plate (Figure 1B ②③); and a fourth comprising the external granule layer (EGL) of the cerebellum (Figure 1B ④); summarised in Figure 1C. In some electroporation experiments (approximately 10%), the boundaries of populations of neurons were obscured by additional ipsilateral axon tracts, including two prominent descending projections (Figure 1D). To determine whether this represented the uptake of DNA by cells outside the rhombic lip, we calibrated our data by constructing a genetic fate map. We co-electroporated a construct with the mouse *Atoh1* enhancer [18], driving the expression of cre-recombinase with a *lox-stop-lox-GFP* plasmid [19]. Under these conditions, additional axon populations are no longer labelled (Figure 1E) confirming the previously identified four populations as derivatives of the *Atoh1*-positive rhombic lip. Any pCAβ-GFP electroporated embryos displaying additional ipsilateral tracts were excluded from our analysis.

### Cell position and axon targets defines nuclear derivatives

**Figure 2:**
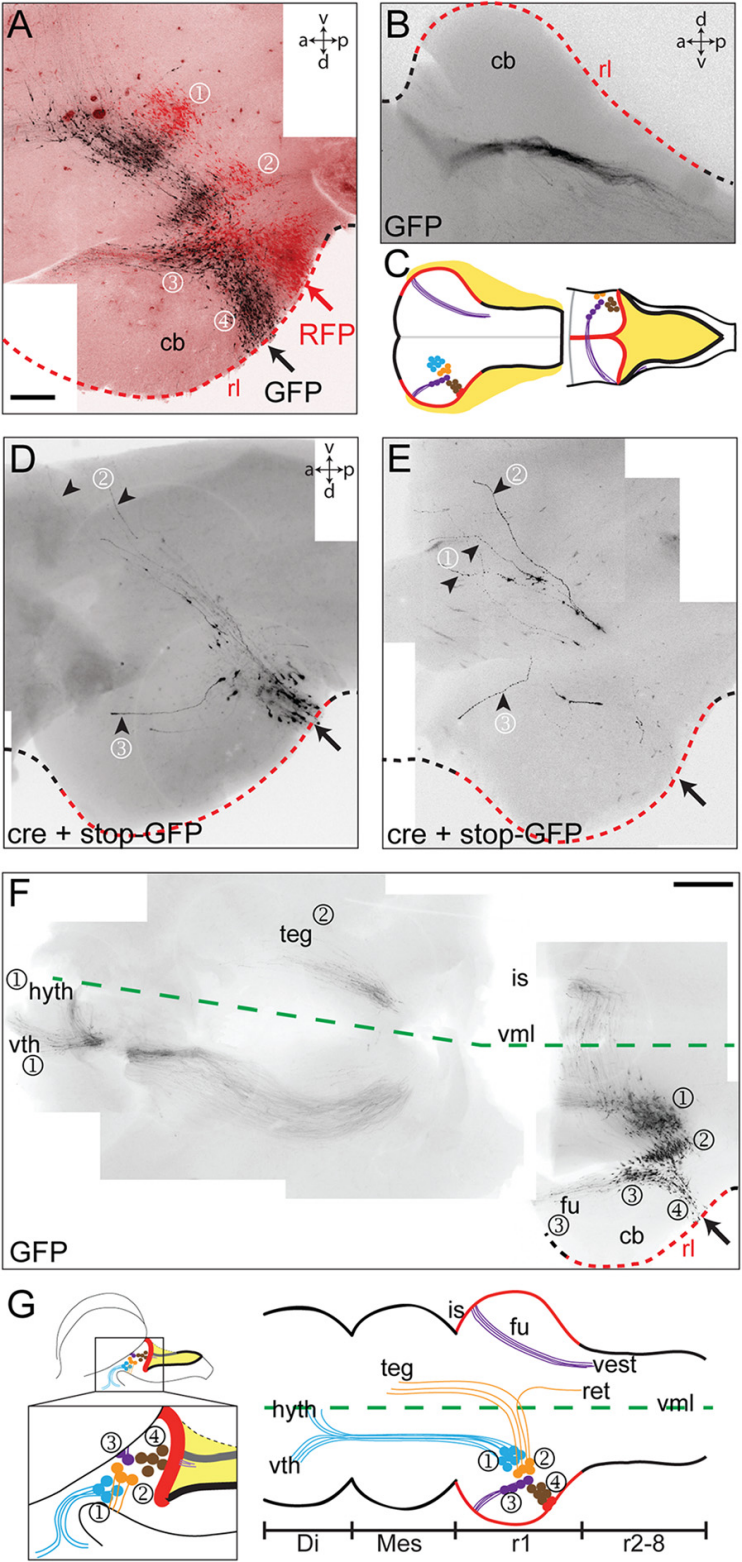
**Axon projections identify populations of rhombic lip derivatives.** Labelled cell populations following electroporation (arrows) into the r1 rhombic lip at st.23/E4 of: **(A)** green fluorescent protein (GFP) and red fluorescent protein (RFP) side-by-side; **(B)** GFP in contralateral fasciculus uncinatus (fu) whose trajectory is schematically represented in **C**; **(D, E)** stop-GFP + diluted (10^-4^) cre to produce a scattered GFP label; **(F)** GFP, shown in a low magnification view of a reconstructed, flat-mounted hindbrain, midbrain and forebrain with the ventral midline (vml) shown as a green dashed line. **G**. Summary diagram of these data showing the relationship between cell groups and their axon targets: hyth, hypothalamus; vth, ventral thalamus; teg, tegmentum; is, isthmus; ret, reticular formation; vest, vestibular nuclei; di, diencephalon; mes, mesencephalon. Scale bars: 200 μm in **A**, **B**, **D** and **E**, 500 μm in **F**.

While the EGL is distinct by its position within the cerebellum, we sought to establish the identity of the remaining three rhombic lip-derived populations on the basis of axon projections and migration patterns. The most dorsal population (③) is distinguished by a specific, rostral cell migration. This is most clearly shown by electroporating GFP and RFP plasmids into adjacent loci at the cerebellar rhombic lip (shown in red and black in Figure 2A). Neurons from the more caudal electroporation integrate with the more rostral stream, but only within the nucleus itself. This suggests that neurons translocate rostrally only once they have reached their target dorsoventral coordinate, following the course of rostrally orientated axons. These axons are likely to derive directly from the leading processes of migrating neurons [20] and extend in a circumferential arc around the boundary of the EGL, across the midline dorsally and then form a descending contralateral pathway in the hindbrain (Figure 2B): the fasciculus uncinatus, shown schematically in Figure 2C. We used a strategy of sparse GFP cell labelling, generated by co-electroporating lox-stop-lox-GFP with a dilute (10^-4^) concentration of pCX-Cre (cre-recombinase), to confirm that only rostrally displaced cells contribute to this axon tract (Figure 2D,E), thus identifying them as neurons of the medial cerebellar nucleus [17]. By contrast, axons derived from the ventrally adjacent cluster at the cerebellar margin (②) project directly towards the ventral midline (Figure 2D,E), while those in an extra-cerebellar position (①) follow a deeper, ventral trajectory but deviate rostrally (Figure 2E). These different initial trajectories in ventrally projecting axons are reflected in their targets, visualised in flat-mounted half brains (Figure 2F). The axons of the extra-cerebellar nuclei (①) rarely cross the ventral midline, but rather form an ascending ipsilateral tract that terminates in two locations within the ventral diencephalon, consistent with this population comprising homologues of the early-born extra-cerebellar rhombic lip derivatives found in mouse [5,6,9]. By contrast, directly ventrally projecting axons from cells at the cerebellar margin (②) cross the midline, turn rostrally and predominantly terminate in the ventral tegmentum. A small crossed population also projects caudally to the contralateral hindbrain (data not shown). These targets are consistent with neurons of the lateral cerebellar nucleus [17], summarised in Figure 2G.

### Rhombic lip cell fate and gene expression are chronotopically organised

**Figure 3:**
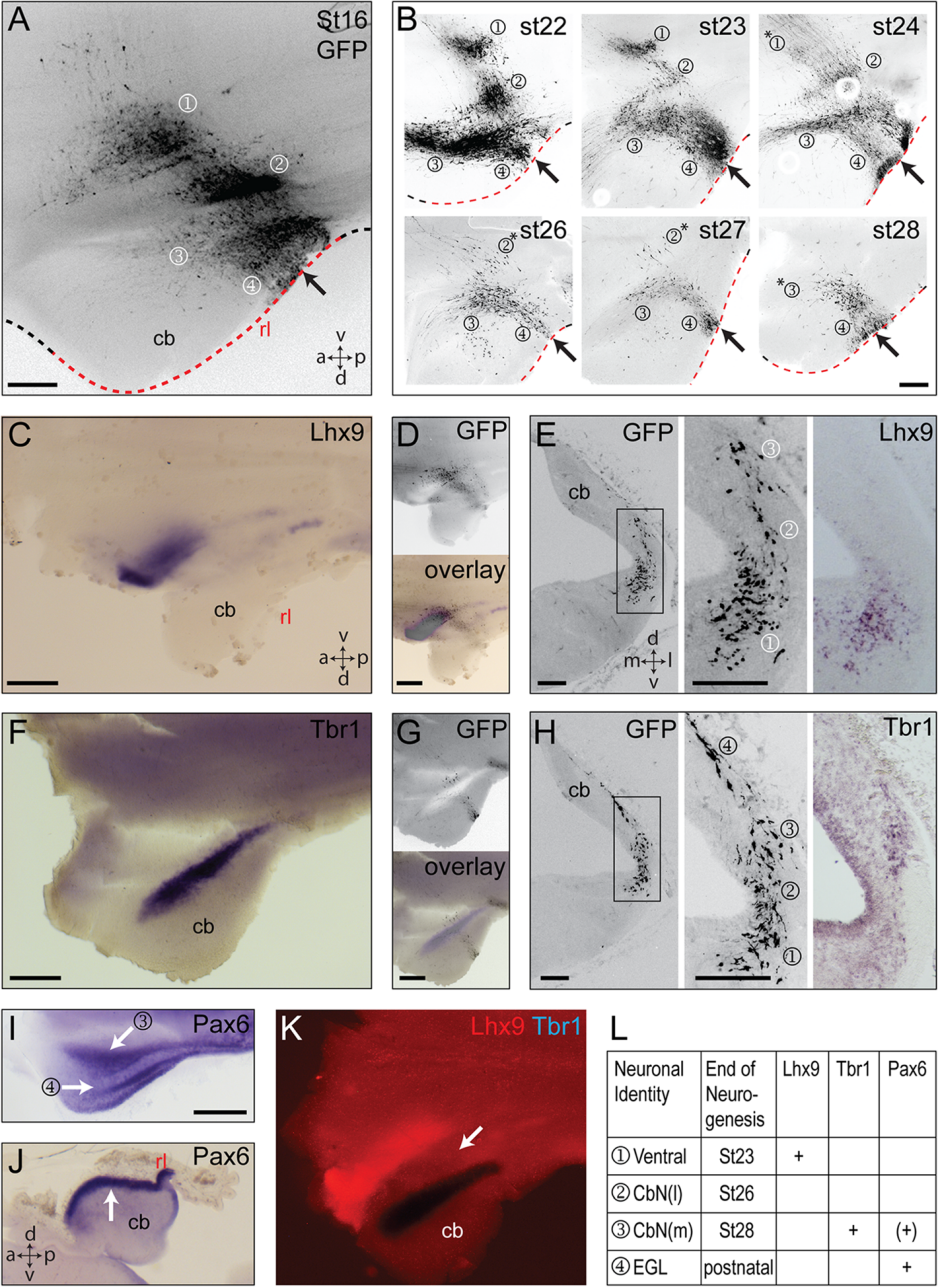
**A temporal sequence of rhombic lip derivatives express *Lhx9*, *Tbr1 *or *Pax6*. A**. Cumulatively labelled cells at st.31/E7 following electroporation of green fluorescent protein (GFP) into the r1 rhombic lip (arrow) at st.16/E3. **B**. Electroporation (arrows) at successively later stages (st.22-st.28) labels progressively fewer populations (①②③④) at st.31 (stage of electroporation indicated top right): *indicates only a residual population. **C**. *Lhx9* expression in a st.31/E7 embryo electroporated with GFP at st.16/E3. **D**. GFP (above) and overlay with *Lhx9* expression (below). **E**. Serial coronal sections through an embryo at st.31/E7 electroporated with GFP at st.23/E4 (left) with higher magnification view of GFP (middle) and *Lhx9* (right) in the most ventral cells (①). **F**. *Tbr1* expression in a st.31/E7 embryo electroporated with GFP at st.26/E5. **G**. GFP (above) and overlay with *Tbr1* expression (below). **H**. Serial coronal sections through an embryo at st.31/E7 electroporated with GFP at st.23/E4 (left) with higher magnification view of GFP (middle) and *Tbr1* (right) in the medial cerebellar nucleus (②). **I**. *Pax6* expression in the medial cerebellar nucleus (③ white arrow) and EGL (④ white arrow) in a flat-mounted E6 brain. **J**. *Pax6* labels only EGL (white arrow) at E8, shown in sagittal Section. **K**. *Lhx9* (red) and *Tbr1* (blue) highlighting gap (white arrow) between expression domains where cells of the lateral cerebellar nucleus reside. **L**. Summary of periods of neurogenesis and gene expression in rhombic lip derived populations: ventral nuclei ①; lateral cerebellar nucleus CbN(l) ②; medial cerebellar nucleus CbN(m) ③; external granule layer (EGL) ④. m, medial; l, lateral. Scale bars: 200 μm in **A-B** and **E-H**, 500 μm in **C-D**, **F-G**, **I-J** and **K**.

To determine the precision of the relationship between cell fate and the timing of neurogenesis, we performed focal electroporations of GFP (n >100) into the cerebellar rhombic lip at each embryonic stage between st.16 (E3) and st.29 (E6) and collected the embryos at st.31 (E7). By comparing the populations excluded from progressively later electroporations, we are able to infer the time at which production of each population terminates in reference to the populations labelled at st.16 (Figure 3A). Thus (Figure 3B), extra-cerebellar cells (①) are labelled through to st.23 with only a scattering visible at st.24. The vast majority of lateral cerebellar nucleus neurons (②) are generated by st.25 while the medial cerebellar nucleus (③) production continues to st.28. Subsequent electroporations (data not shown) label only the EGL (④). We then contrasted GFP labelling directly with markers of neuronal identity that have been established in the mouse using *in situ* hybridisation: *Tbr1*, a marker of the fastigial cerebellar nucleus in mouse [7]; *Lhx9*, a marker of dentate/interposed cerebellar nuclei and all extra-cerebellar rhombic lip derivatives in r1 [9,21]; *Pax6*, expressed in late-born derivatives.

*Lhx9* is expressed exclusively in the most ventral extra-cerebellar neurons (Figure 3C), as confirmed by comparison with GFP labelling at E3 and E4, in whole-mount (Figure 3D) and in coronal sections (Figure 3E), respectively. *Tbr1* is expressed in a stripe at the edge of the cerebellum (Figure 3F), which comparison with GFP electroporations at E5 and at E4 in whole-mount (Figure 3G) and coronal section (Figure 3H), respectively, reveals as the medial cerebellar nucleus. *Pax6* is also expressed in the medial cerebellar nucleus at E6 (Figure 3I), but, as in mice, [7], becomes restricted to the EGL at later stages (E8: Figure 3J). Lateral cerebellar nucleus neurons lie within an *Lhx9*-negative, *Tbr1*-negative, *Pax6*-negative domain (Figure 3K). Thus, different periods of neurogenesis are correlated with the expression of characteristic genes (Figure 3L).

### *Lhx9* expression differentiates chicks from mice

**Figure 4:**
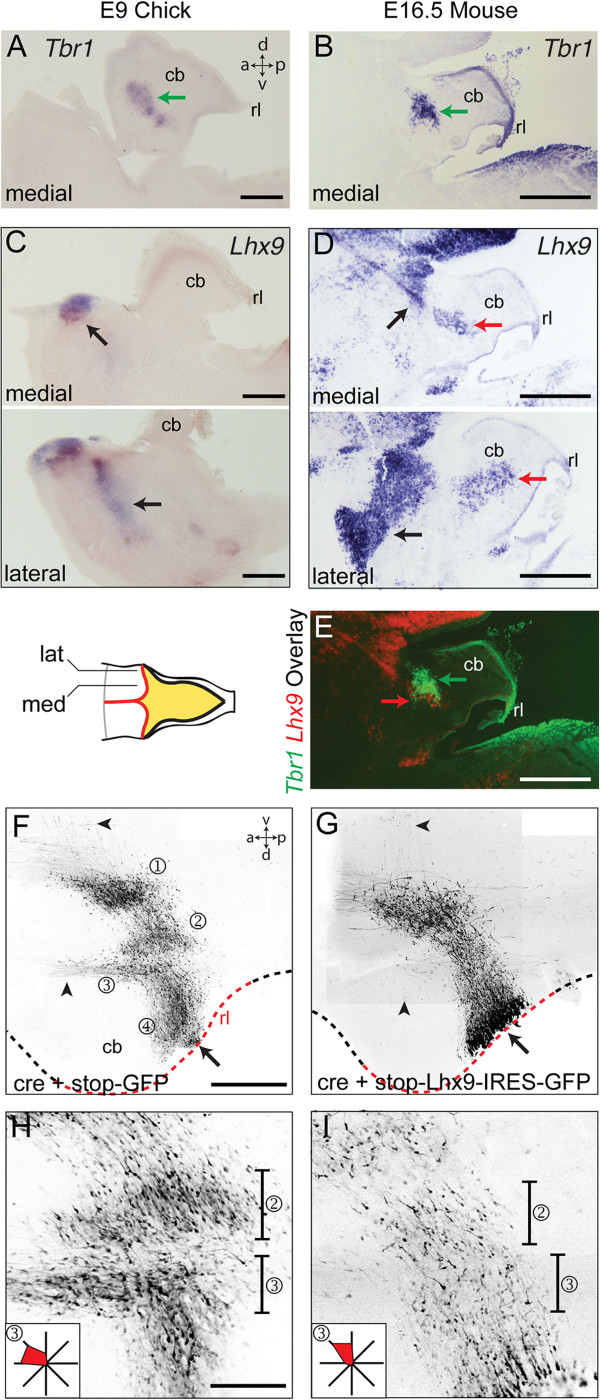
***Lhx9 *overexpression in chicks alters nucleogenesis and initial axon trajectory of rhombic lip derivatives. A**, **B**. Medial/fastigial nucleus expression of *Tbr1* in sagittal sections of st.35/E9 chicks **(A)** and E16.5 mice **(B)**. **C**. *Lhx9* expression in medial (above) and lateral (below) sagittal section of st.35/E9 chicks in extra-cerebellar populations (black arrows). Since these are lateral sections, the area of cerebellar tissue is smaller than mid-sagittal sections. **D**. *Lhx9* expression in medial (above) and lateral (below) sagittal section of E16.5 mouse in both extra-cerebellar populations (black arrows) and dentate nucleus (red arrows). **E**. Overlay of **B** and **D** showing close apposition of *Tbr1* (green arrow) and *Lhx9* (red arrow) labelled neurons in cerebellar nuclei. **F**, **G**. Flat-mounted embryos at st.31/E7 electroporated at st.24/E4 with either cre + stop-GFP **(F)** or cre + stop-Lhx9-IRES-GFP **(G)**. Arrowheads indicate the position of axons from cerebellar nuclei **(F)**, which are absent following *Lhx9* overexpression **(G)**. Single optical sections shown respectively in **H** and **I** show lateral (②) and medial (③) cerebellar nuclei at high magnification with vectors of initial cell processes (in region ③) shown inset as relative frequencies on a radial plot. Scale bars: 500 μm in **A-E** and **F-G**, 200 μm **H-I.**

Mouse and chick cerebellar nuclei differ in both their complement and connections. Notably, the dentate nucleus, which has a projection to the dorsal thalamus, appears to be a mammalian innovation. To examine developmental differences between chick and mouse cerebellar anatomy, we first contrasted the expression of *Tbr1* and *Lhx9* markers in chicks at E9 and mice at E16.5. *Tbr1* defines the medial/fastigial cell group in both chicks (Figure 4A) and mice (Figure 4B). However, whereas *Lhx9* expression in chicks is restricted to extra-cerebellar neurons (Figure 4C), in mice, *Lhx9* also labels neurons within the cerebellar white matter (Figure 4D) corresponding to the dentate nucleus. Superimposition of the expression of *Tbr1* and *Lhx9* in E16.5 mouse brain (Figure 4E) shows a correspondingly narrowed spatial gap between *Lhx9* expression and *Tbr1* in mice compared to chicks. This suggests that the window of *Lhx9* expression in relation to the sequence of cell production at the rhombic lip is extended in mice compared to chicks.

To explore whether the species-specific differences in *Lhx9* expression are sufficient to account for altered axon targeting of cerebellar nuclei, we co-electroporated pCX-Cre into the E4 chick rhombic lip with a lox-stop-lox-Lhx9-IRES-GFP [14]. Compared to controls (lox-stop-lox-GFP: Figure 4F), the dorsoventral migration of cells expressing ectopic *Lhx9* is normal (Figure 4G). However, the distinction between nuclei (①②③④) was abolished with ventral contralateral axon projections and the dorsal fasciculus uncinatus (arrowheads) absent in all cases (n = 12). Correspondingly, the rostral displacement of medial cerebellar neurons (③) was absent in *Lhx9* expressing cells (Figure 4G). A comparison of GFP labelling in single confocal optical sections of control (Figure 4H) and *Lhx9* electroporated embryos (Figure 4I) in the region of the cerebellar nuclei shows that the initial segments of all axons are orientated ventrally (vector plots, inset). Since no additional projections were seen to cross the midline (Figure 4G) axons of cells expressing up-regulated *Lhx9* were excluded from all but an ascending ipsilateral axon tract directed towards the diencephalon.

## Discussion

In this study we have shown the origins of the two cerebellar nuclei of birds at the rhombic lip and explored their temporal molecular patterning. Differences between *Lhx9* expression in chicks and mice reflect the different organisation of their respective cerebellar projections. Furthermore, *Lhx9* overexpression is sufficient to re-specify axon initial trajectory but not tangential migration of chick rhombic lip derivatives.

### Timing, neurogenesis and nucleogenesis in rhombic lip derivatives

Our results reinforce the overall principle that the dorsoventral position of rhombic derivatives is correlated with the organisation of discrete neurogenic temporal windows [5,6,22,23]. Patterning of cell fate is likely to be autonomous to the rhombic lip itself and we show that the precision of timing extends to the subdivisions of closely apposed cell groups within the NTZ. Medial and lateral cerebellar nuclei become progressively, spatially differentiated in a process of nucleogenesis that we demonstrate is dependent on short-range axonopetal movements. These adjustments in cell position are superimposed on a scaffold of dorsoventral positional allocation that is likely to depend on differences in Robo-mediated responses to diffusible midline generated guidance cues [22,24,25]. When the initial segments of all axons are aligned following *Lhx9* overexpression, nuclear boundaries disappear. This suggests that cerebellar nucleogenesis is an epiphenomenon of the autonomous behaviour of individual cells rather than a property of the nucleus *per se*.

### The origin of diversity in cerebellar nuclei

Superimposed on the dorsoventral array of rhombic lip migrants are patterns of gene expression that, by implication, also follow a temporal sequence. Some genes no doubt regulate responses to migratory cues: for example, the migration of rhombic lip derivatives in both the hindbrain and cerebellum is profoundly influenced by *Hox* gene expression [25,26]. However, the overexpression of *Lhx9* leaves cell distribution unaltered along the dorsoventral axis and selectively respecifies axon trajectories, consistent with models that propose an evolutionarily conserved role for LIM-homeodomain proteins in axonogenesis [14,15,27]. This contrasts with LIM-homeodomain gene knock-down studies where, for example, tangential neuronal migration may be specifically attenuated [28].

This observed separation of LIM-independent migration and LIM-dependent axonogenesis programmes provides an attractive explanation for the origin of nucleus diversity. We show that when compared to chicks the duration of expression of *Lhx9* in mice is extended into the window of cerebellar nucleus production. Thus, in contrast to birds, *Lhx9* expression ends as the production of *Tbr1*-positive medial/fastigial neurons commences, implying a specific heterochronic shift in gene expression. This results in the generation of a lateral (dentate) nucleus expressing *Lhx9* that, in common with extra-cerebellar rhombic lip derivatives, targets relay neurons of the thalamus. While our overexpression data are consistent with this hypothesis, we were unable to determine whether chick axons from cells expressing ectopic *Lhx9*-target the thalamus. Similarly, we were unable to detect contralateral ascending projections from this transformed nucleus, suggesting that additional factors might be required to fully recapitulate a dentate-like contralateral axon trajectory in chicks.

The significance of the dentate nucleus in mammalian evolution is in allowing cortical function to be modulated by the cerebellum. However, this is only possible if cortical output is also relayed to the cerebellum via mossy fibre projecting pontine neurons to complete a closed-loop cortico-cerebellar circuit [1]. In the absence of the dentate nucleus, the pontine nucleus in birds is, perhaps unsurprisingly, small [29,30] with no reported analogue of the prominent anterior extramural migration stream of contributing neuroblasts seen in mammals [31]. Evolution of the circuit thus appears to have required a co-adaptation in both cerebellar nucleus specification and pontine organisation and scale. Both pontine neurons [25,32,33] and cerebellar nuclei are derivatives of the rhombic lip, but from spatially remote rostral and caudal precursor pools, respectively. Hence, evolutionary changes are likely to be downstream of coordinated adaptation of both the hindbrain and cerebellar rhombic lip.

## Conclusions

The production of cerebellar nuclei in birds and mammals are similarly organised along temporal cues; however, birds lack a thalamic projecting dentate-like nucleus. This confirms the presence of discrete neurogenic temporal windows for rhombic lip derivative cell fate. Our results suggest that *Lhx9* may be a key determinant of thalamic connectivity. Evolution of mammalian connectivity may thus reflect adaptation in the timing of *Lhx9* expression with respect to fate allocation of rhombic lip derivatives. This points to the importance of temporal patterning in the formation of the cerebellar circuit and predicts that coordinated changes in pontine rhombic lip derivatives were required for the formation of the corticocerebellar circuit.

## Methods

### *In ovo* electroporation

Fertilised wild type HiSex chicken eggs (Henry Stewart, Louth, Linconshire, UK) were incubated at 38°C for three to six days prior to windowing with sharp surgical scissors. The fourth cranial ventricle was injected with 100 to 200 nl of 0.8 to 2 μg/μl DNA plasmid, singly or in combination: pCAβ-RFP, pCAβ-eGFP-m5 [34], Atoh1-cre [19], pFlox-pA-EGFP (lox-stop-lox-GFP) and pCX-Cre [35], lox-stop-lox-Lhx9-IRES-GFP [14]. Three 20 ms/10 V square waveform electrical pulses were passed between electrodes placed on either side of the hindbrain, targeting the rhombic lip of rhombomere 1 (Figure 2A). Eggs were resealed and were incubated for a further one to four days at 38°C.

### Histology and photomicroscopy

Electroporated chick embryos and control chick and CD1 mouse embryos were fixed in 4% (w/v) paraformaldehyde (in phosphate-buffered saline) and either dissected or processed for cryostat sectioning. Tissue was stained by *in situ* hybridisation (Myat *et al*., 1996) with digoxygenin-labelled (Roche Diagnostics Limited, Burgess Hill, West Sussex, UK) riboprobes for (mouse) *Lhx9*, *Tbr1* (Alessio Delogu, Kings College London, UK) or (chick), *Lhx9* (Alessio Delogu), *Tbr1*[30] and *Pax6*[36]. Following *in situ* hybridisation tissue was processed for fluorescence immunohistochemistry using an anti-GFP antibody (IgG 1:100, Invitrogen). Digital images were acquired on either stereo (Leica MZFLIII) or compound (Nikon Elipse80i) microscopes equipped with epifluorescence or by laser scanning confocal microscopy (Olympus AX70). Axon initial segment orientation was measured in ImageJ.

## Abbreviations

EGL: External granule layer; GFP: Green fluorescent protein; NTZ: Nuclear transitory zone; r: Rhombomere; RFP: Red fluorescent protein.

## Competing interests

The authors declare that they have no competing interests.

## Authors’ contributions

MJG and RJTW planned the study and prepared the manuscript. MJG performed the experiment and analysis. Both authors read and approved the final manuscript.
